# *Yersinia ruckeri*, an unusual microorganism isolated from a human wound infection

**DOI:** 10.1002/nmi2.56

**Published:** 2014-06-19

**Authors:** S De Keukeleire, A De Bel, Y Jansen, M Janssens, G Wauters, D Piérard

**Affiliations:** 1 Department of Microbiology and Infection Control, Universitair Ziekenhuis Brussel, Vrije Universiteit Brussel (VUB)Brussels, Belgium; 2 Department of Surgery, Universitair Ziekenhuis Brussel, Vrije Universiteit Brussel (VUB)Brussels, Belgium; 3 National Reference Centre for Yersinia, Secteur des sciences de la santé – Pôle de Microbiologie Médicale, Université Catholique de LouvainBrussels, Belgium

**Keywords:** Human, MALDI-TOF MS, wound infection, *Yersinia ruckeri*

## Abstract

We report the first documented case of *Yersinia ruckeri* isolated from a wound infection, in a 16-year-old male after hitting a stone while paddling in a river.

## Introduction

An adolescent male consulted his general physician to suture a deep wound of his left lower leg (day −1) after hitting a stone while paddling in a river in the Ardennes; a region in the southeast of Belgium. The next day, he presented at the Emergency Department for evaluation of the wound (day 0). The lesion felt warm, painful and was accompanied by cellulitis. The patient had no fever. Laboratory findings showed a slightly raised level of C-reactive protein of 14.5 mg/L (reference value <5 mg/L) and a white blood cell count of 12.9 × 10^3^/mm^3^ with an absolute neutrophilia of 8.462 × 10^3^/mm^3^ (reference value 1.4 × 10^3^ to 6.7 × 10^3^/mm^3^). No systemic complications were recorded. A gauze wick soaked in topical antiseptic solution (povidone-iodine) was placed after the abscess was opened and intravenous amoxicillin–clavulanic acid (4 × 1 g/day) was started as empirical antibiotic therapy.

Wound swabs were cultured on MacConkey agar, Trypticase Soy Agar-based medium enriched with 5% horse blood with 20 mg/L haemin (Hem side) or 40 mg/L nalidixic acid (NaI side) (Hem/Nal agar) and on an anaerobic agar enriched with 5% defibrinated horse blood. Identification took place using matrix-assisted laser desorption ionization time-of-flight mass spectrometry (MALDI-TOF MS; Microflex, Bruker Daltonik GmbH, Bremen, Germany) by direct transfer method and in combination with database version 3.3.1.0 (Bruker, Daltonik GmbH) and security relevant (SR) database. 16S rRNA sequencing was performed at the National Reference Centre for *Yersinia* using the method described by O'Neill *et al*. 1,2. Phenotypic characteristics were determined using conventional methods 3. Antibiotic susceptibility was performed on Mueller–Hinton agar by the EUCAST methodology.

After 24 h of incubation, cultures of the infected wound yielded a mixed culture. MALDI-TOF MS analysis of each colony type showed the presence of *Aeromonas* spp., *Lactobacillus* spp., *Clostridium perfringens* and *Yersina ruckeri*. The latter showed small (1-mm diameter) non-pigmented circular colonies on MacConkey agar and Hem/Nal agar and flat to convex colonies on anaerobic agar. Growth was optimal between 25 and 30°C. The MALDI-TOF MS log score was: best match *Y. ruckeri* 1.969 and second-best match *Yersinia pestis* (log score 1.887). *Yersinia pseudotuberculosis* came out as third species on the sixth place (log score 1.726). Identification was confirmed by the National Reference Centre *Yersinia*. 16S rRNA gene sequence analysis of the strain revealed the highest similarity (100%) to *Y. ruckeri* strain PIC 8 and 99.8% similarity to *Y. ruckeri* type strain (ATCC29473).

The strain fitted the conventional phenotypic characteristics of Enterobacteriaceae spp., e.g. oxidase-negative, catalase-positive fermenting gram-negative rods. Glucose and mannitol were acidified without gas production, but sucrose, xylose, rhamnose and melibiose were negative. Urease, esculin hydrolysis, indole and H_2_S production and Voges–Proskauer reaction were negative. Simmons' citrate was delayed positive. In contrast to almost all other *Yersinia* species, lysine decarboxylase and gelatine hydrolysis were positive 3–5. Antimicrobial susceptibility testing showed sensitivity to ampicillin, amoxillin/clavulanic acid, piperacillin/tazobactam, temocillin, cefazolin, cefuroxime, ceftriaxone, ceftazidime, ceftazidime/clavulanic acid, cefepime, cefepime/clavulanic acid, meropenem, aztreonam, gentamicin, amikacin and ciprofloxacin. At day +3 the patient could be discharged from the hospital with daily local wound care and an equivalent oral antibiotic regimen. At follow-up consultations at day +10 no signs of infection were present, so local treatment was switched to local wound care and antibiotics were discontinued. At consultation +20 complete healing of the wound was observed.

Curiously, *Y. ruckeri* was first described as the causative agent of yersinosis or enteric red mouth disease, which affects mainly salmonid fish 6–11. Infections due to *Y. ruckeri* cause high mortalities in fish aquaculture systems, especially in rainbow trout (*Oncorhynchus mykiss*), leading to significant economic losses in the fish farming industry 9,11,12. The bacterium is shed in the faeces of infected fish and the disease can be transmitted by water. *Yersinia ruckeri* is able to survive for long periods of time (more than 4 months), especially after an outbreak of the disease 10,13. *Yersinia ruckeri* remains infective in an aquatic environment, mainly associated with poor water quality 11,12. The strain has the ability to adhere on solid surfaces and to form biofilms 12,14. This case report underlines the high discriminative power of MALDI-TOF MS. The potential pathogenicity of *Y. ruckeri* in human wound infections remains unanswered and further evaluation is needed. In this case it remains unclear whether *Y. ruckeri* or another bacterium (*Aeromonas* spp., *Lactobacillus* spp. or *Clostridium perfringens*) caused the infection. Nevertheless, to the best of our knowledge, isolation of *Y. ruckeri* from human wounds or biopsies and its possible association with infection in humans was never previously described.
